# A Model for the Determination of Pollen Count Using Google Search Queries for Patients Suffering from Allergic Rhinitis

**DOI:** 10.1155/2014/381983

**Published:** 2014-06-19

**Authors:** Volker König, Ralph Mösges

**Affiliations:** Institute of Medical Statistics, Informatics and Epidemiology, University of Cologne, 50924 Cologne, Germany

## Abstract

*Background*. The transregional increase in pollen-associated allergies and their diversity have been scientifically proven. However, patchy pollen count measurement in many regions is a worldwide problem with few exceptions. *Methods*. This paper used data gathered from pollen count stations in Germany, Google queries using relevant allergological/biological keywords, and patient data from three German study centres collected in a prospective, double-blind, randomised, placebo-controlled, multicentre immunotherapy study to analyse a possible correlation between these data pools. *Results*. Overall, correlations between the patient-based, combined symptom medication score and Google data were stronger than those with the regionally measured pollen count data. The correlation of the Google data was especially strong in the groups of severe allergy sufferers. The results of the three-centre analyses show moderate to strong correlations with the Google keywords (up to >0.8 cross-correlation coefficient, *P* < 0.001) in 10 out of 11 groups (three averaged patient cohorts and eight subgroups of severe allergy sufferers: high IgE class, high combined symptom medication score, and asthma). *Conclusion*. For countries with a good Internet infrastructure but no dense network of pollen traps, this could represent an alternative for determining pollen levels and, forecasting the pollen count for the next day.

## 1. Introduction

Allergic rhinitis (AR), together with the comorbidity of asthma, ranks among the most frequent chronic diseases of our time [1, 2]. Its prevalence rates continue to rise and are thereby defining it as a global health problem. In several countries, the prevalence rate of AR in young adults is over 40% [2]. In the therapeutic ranking the avoidance of the allergen exposure comes first. An already established measurement supporting the avoidance of pollen exposure of affected persons suffering from seasonal AR is the measurement of the pollen count on the basis of pollen traps [3, 4]. This is especially drawing attention to the strength of the pollen count. Forecasting and providing the patients suffering from AR with these forecasts allow them to take measures to specifically avoid either strong or any pollen exposure. Possible measures could be an airing according to the time of the day and a postponement of outdoor activities. The warning of a high pollen count furthermore enables the patients to prepare themselves these days by taking along symptomatic medication especially for asthmatics.

Data from clinical trials on AR must be interpreted in the context of pollen exposure. Only when it is ensured that the patients were in fact exposed to the allergens of their individual pollinosis, can it be assumed that therapy has induced symptom improvement. In placebo-controlled double-blind trials, it has therefore also become standard procedure to determine regional pollen concentrations. Based on such data it is possible to detect a more precise contrast between the symptoms of the placebo group and those of patients receiving active treatment. One requirement for this is a frequent documentation of local pollen concentrations, as is the case in Kuwait [5]. In countries with larger areas, it encounters limits since the number of stations needed can quickly reach magnitudes that are no longer affordable. Furthermore, it must be considered that each type of pollen differs in terms of its travel duration, dispersal distance, and time/period of release. Birch pollen grains, for example, can be carried over 1000 km, while grass pollen usually does not travel more than 100 km [6–9]. With few exceptions [5], it is precisely the patchy pollen count coverage in many regions that poses a worldwide problem. Even the highly financed network of German pollen count stations is not complete, with distances of more than 100 km between the counting stations. In the vast expanses of the USA, the method of pollen measurement reaches its limits entirely. Alternatives, such as those in which each participant in a clinical trial is equipped with an individual allergen sampler, have not been able to assert themselves [10].

In 2008, the company Google launched Google Insights for Search, an Internet-based information service that displays all queries (keywords) entered by users in the Google search engine in terms of numbers and regions. The time- and region-stamped queries could help support the previous pollen count measurement and also analytically derive a denser network of pollen count analysis. At the end of September 2012, Google Insights for Search was integrated into Google Trends and continues to be available as an analysis tool.

We have recently found that regionally measured pollen concentrations and Google queries using allergy-typical search terms exhibit a similar time period [11]. Several investigations, although unrelated to AR, have already been published on the use of these modern, Internet-based source data from Google Insights for Search or Google Trends [12–16].

The aim of the analyses described below was therefore to examine whether the frequency of Google searches for allergy-typical terms correlates similarly well with the disease severity of patients with pollen-induced AR, as it has been assumed for regionally measured pollen concentrations and verified in numerous studies [17, 18].

## 2. Methods

**Figure 1:**
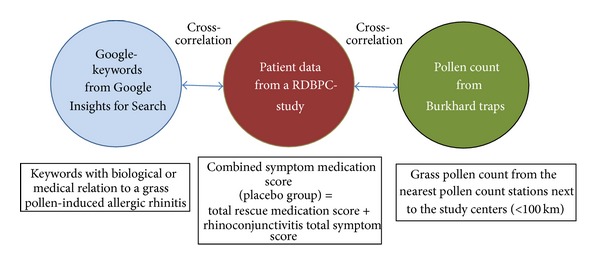
The three data pools.

Figure 1 gives a rough overview on the methods for the correlation analysis of the data pool used, which is described in the following section. Besides, the middle circle represents the gold standard for the analysis, defined by the pooled combined symptom and medication score from patients of a randomized, double-blind, and placebo-controlled (RDBPC) clinical study.

### 2.1. The Clinical Study

As part of this investigation, a prospective, double-blind, randomised, placebo-controlled, multicentre subcutaneous immunotherapy study was examined with regard to a correlation between the pollen count at the time and the symptom-medication values of the patients (placebo group). This thirteen-month study included patients aged 18 to 75 years who had the clinical picture of AR and/or allergic rhinoconjunctivitis with/without seasonally controlled allergic asthma. These patients had to be sensitised to grasses (positive prick test for grass, wheal diameter > 3 mm), and AR symptoms had to appear primarily during the grass pollen season. Furthermore, the 18-level total symptoms sum score in RRTSS (retrospective rhinoconjunctivitis total symptom score, 0: no symptoms; 18: maximal symptoms) [19] of the respective patient had to be ≥8 in the previous pollen season. An exclusion criterion was the predominance of perennial AR. Moreover, the patients were required to keep a diary in which they were to record their symptoms and possible consumption of concomitant medication on a daily basis. Entries were limited to the months of May, June, and July 2009. These diary data are the basis for this investigation.

Overall, three study centres participated in the study ((1) Aachen/German state of North Rhine-Westphalia (NRW), (2) Hamburg/German state of Hamburg, and (3) Wiesbaden/German state of Hesse). Subsequent analyses were only performed for the placebo group to rule out drug-related biases.

The three cohorts were then examined with respect to the rhinoconjunctivitis total symptom score (RTSS 0–3, symptoms: sneezing, runny nose, itchy nose, nasal congestion, tearing, and itchy eyes) and the total rescue medication score (TRMS: chromones (eyedrops) = 1 point, antihistamine (oral) = 2 points, corticosteroids (nasal) = 3 points, on demand: leukotriene antagonists = 5 points). Afterwards, both scores were added together to a combined symptom medication score (CSMS) to compare the data with the Google data and the pollen count data.

Four subgroups were formed for the three cohorts in order to more exactly interpret the significance of the strengths of the correlations with pollen counts.

*Subgroup 1* (Patient Cohort according to Average CSMS). The basis of calculation in this group was the averaged CSMS. The scores of all patients from one centre were added together and the mean was generated.

*Subgroup 2* (Determining the IgE Classes). Besides the total IgE value, the specific IgE values were analysed for the single allergen timothy grass in this study. Together with the clinical picture (inclusion criteria in the study: positive history and skin prick test for grasses), this suggests a grass allergy in a patient [20]. Two subgroups were formed according to the grass pollen-specific IgE classes of “IgE low” (0: <0.35/I: 0.35–0.69/II: 0.70–3.49) to “IgE high” (III: 3.50–17.49/IV: 17.5–49.99/V: 50–100/VI: >100).

*Subgroup 3* (Comorbidity-Asthma). In this analysis, the patients from all centres were divided into “asthma” and “no asthma” groups.

*Subgroup 4* (Grouping according to CSMS). Patients were divided into the two groups “CSMS high” and “CSMS low.” The basis for allocating the patients to the respective groups was the calculation of the group-specific mean CSMS of the respective centre. The patients CSMS in the “CSMS low” group were below 50% of the calculated mean of the respective patient population of the individual centres.

### 2.2. Keyword Analysis

To find appropriate terms for a suitable correlation, all terms of interest to a pollen allergy sufferer were investigated. In doing so, general terms such as “allergy” or “pollen count” and more specific allergological terms were of interest, such as the names of respective drugs most frequently requested in Germany.

The first step involved entering the search terms in the “Google AdWords” keyword tool (Step 1). This instrument displays search terms according to number and the location (state) chosen. This step investigates whether the keywords were entered in Google using the same or similar syntax. This tool also suggests synonyms, related words, or word combinations, which would be an interesting aspect for further analysis (Step 2).

Another option for identifying suitable keywords is “Google Correlate.” This is an analysis tool that outputs queries correlating best with the entered keyword. In this case, the term “hay fever” was investigated, since it constitutes the strongest medical correlation. The strongest 20 terms were included for further analyses.

Step 2 consisted of entering the identified keywords into the tool “Google Insights for Search.” In addition to the simple search of the identified keywords, the keywords were also entered in combination. In this step, all terms were excluded for which there was no sufficient frequency and therefore no data in the patient diary. Insufficient data and hence the exclusion for the following analyses are based on the fact that the output of “Google Insights for Search” only includes keywords or combinations for popular search terms and does not depict terms with low volume. The absolute number or the limits of the search requests are not mentioned by Google. The data put out by Google for the requested period and region are normalised on a scale from 0 to 100. Repeated queries from a certain user over a short period of time were eliminated.

#### 2.2.1. Overlap of Birch and Grass Pollen Count

**Figure 2:**
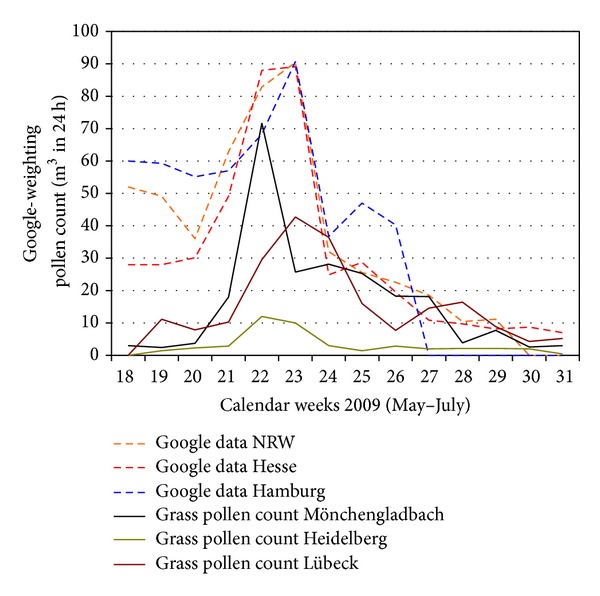
Courses of Google data depicted on the exemplary keyword “hay fever” versus grass pollen count from the pollen count stations.

**Figure 3:**
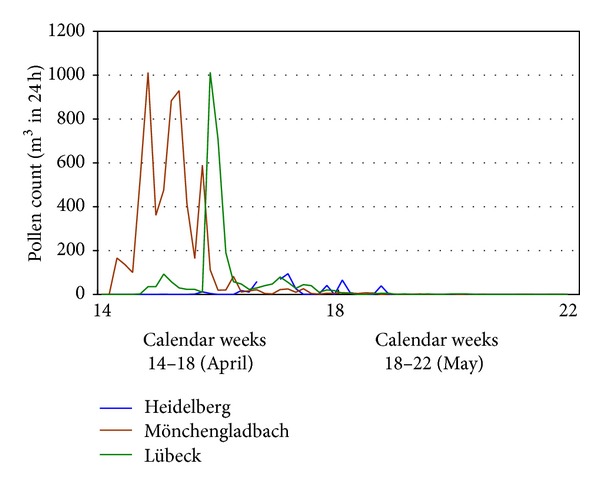
Birch pollen count, April-May 2009.

Obviously, other levels of baseline values were recognised in the Google data than in the actual pollen count data (grasses). Real pollen counts begin at zero in May and peak once before subsiding at the end of July. The Google data, on the other hand, start at a weighting of 30–60% and, except for Hamburg, subside similarly after peaking once (Figure 2). The reason for this is the effect of birch pollen up to the beginning/middle of May in connection with keyword searches not specific to the grass pollen allergy (Figure 3). For the subsequent correlation analyses, weighting of the Google data was therefore adjusted in a second step with the pollen counts (birch, travel distance up to 1,000 km) from the separate counting stations, to minimise the influence of the birch pollen count. These values were displayed and analysed as “Google data (2).” The basis of the calculation was the division of the grass pollen count data by the pooled data of the birch and grass pollen counts on the respective day in the observation period. Afterwards, the Google data (1) were adjusted according to the resulting values. The numbers of Google data in the figures reflect how many searches have been done using the identified keyword or the keyword-combination, relative to the total number of searches done on Google over the time period from May to July 2009.

### 2.3. Statistics

All analyses were performed using the SPSS statistics software package by IBM (version 20). For data consistency, descriptive analyses were conducted to identify possible duplicates or values outside the norm.

Possible relationships were measured by cross-correlation analysis. This statistical procedure measures the correlation between two time series and has the advantage of calculating such correlations under consideration of a time shift. The term “lag” used here indicates the shift of days on which the respective cross-correlation coefficient (CCF) value was measured. The CCF reflects the strength of the correlation (0 < CCF < 0.5 = weak correlation, 0.5 ≤ CCF < 0.8 = moderate correlation, 0.8 ≤ CCF ≤ 1.0 = strong correlation). Besides the correlation coefficient and the lag, the standard deviation (SE: standard error) was also calculated. Additionally, the correlation strengths were examined with regard to their two-sided significance.

## 3. Results

After carrying out all investigations in the aforementioned steps, the keywords and combinations of these keywords were analysed for each centre via cross-correlation analysis using the data from Subgroup 1 (patient cohort according to average CSMS). Here, a combination of several keywords proved particularly suitable for further analysis. Hence, only this combination is introduced further in this work, which is also fulfilling the following criteria:

- sufficient data available for all three German states,
- a clinical or biological relationship to grass pollen count or pollinosis,
- long-term significance of the keywords.

The long-term significance of the keywords is of special importance with regard to future analyses in this area, irrespective of location and time. In contrast, there are drug names, for example, that can be replaced over time by more modern products (new generation antihistamines).

### 3.1. Cohort 1 (Aachen)

In this centre, 16 patients in total received placebo. The cities of Aachen (study centre), Bonn (pollen count station, 71.49 km from the study centre), and Mönchengladbach (pollen count station, 52.55 km from the study centre) are located in the state of NRW. Therefore, the Google data from NRW using the respective keywords were compared with the patient data from Aachen.

No asthma patient was treated in this patient population. Thus, there are no investigations for Subgroup 3.

**Figure 4:**
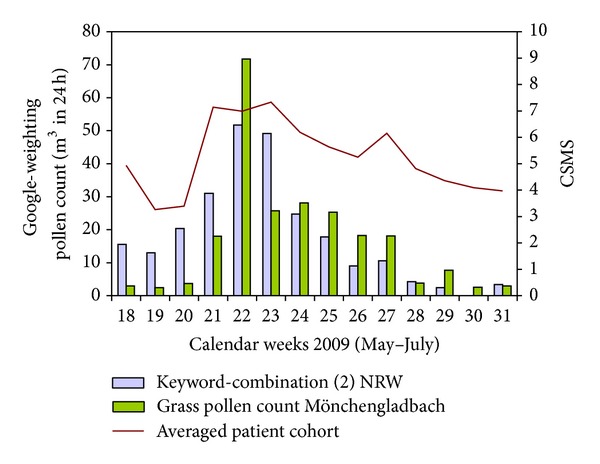
Course for Subgroup 1 (Aachen study centre).* Legend*: the averaged weekly values from Google (“Search Volume Index”) and the pollen count data (pollen count/m^3^ in 24 h) are presented together on the *y*-axis for the sake of clarity. Scaling of the averaged weekly CSMS of the patients is displayed on the opposite axis.

In Subgroup 1, the best values were determined for this patient cohort (Table 1). The grass pollen data for Bonn had a CCF of 0.466 (lag = −1, *P* < 0.001) and 0.630 (lag = 0. *P* < 0.001) for the data from Mönchengladbach compared to the CSMS of the patients. The keyword-combination (2) indeed showed stronger correlations; the value here was at a CCF of 0.715 (lag = −1, *P* < 0.001).  Figure 4 depicts the corresponding course for this group.

### 3.2. Cohort 2 (Hamburg)

In the Hamburg centre (state of Hamburg), a total number of 14 patients received placebo. Valid data were available for analysis from Lübeck (pollen count station, 57.77 km from the study centre) in the neighbouring German state of Schleswig-Holstein. Google data from the state of Hamburg could be analysed for Cohort 2.

Unlike in the Wiesbaden and Aachen study centres, two peaks were observed for the Hamburg patient cohort at the end of May and June, respectively. The IgE value was missing for one patient who was consequently excluded from Subgroup 2 analysis.

The correlation for the averaged patient cohort with the keyword-combination (1) was a CCF = 0.524. For the grass pollen data from Lübeck, only a weak correlation of 0.389 CCF (lag = 0) was calculated for the averaged cohort.

On the other hand, the “IgE high” (0.536) and “CSMS high” (0.557) groups each showed the strongest correlations for the keyword-combination (1). No CCF over 0.5 was observed for the “IgE low” and “CSMS low” groups.

The grass pollen count data from Lübeck did not exceed a CCF of 0.5 in any group. The best value here was a CCF of 0.417 (lag = 0) in the “no asthma” group.

### 3.3. Cohort 3 (Wiesbaden)

The Wiesbaden study centre is located in the state of Hesse. The nearest pollen count station was located in Heidelberg (82.32 km from the study centre) in the neighbouring state. Data directly from the state of Hesse were used for the Google-specific analysis. Overall, 22 patients from this centre received placebo.

**Figure 5:**
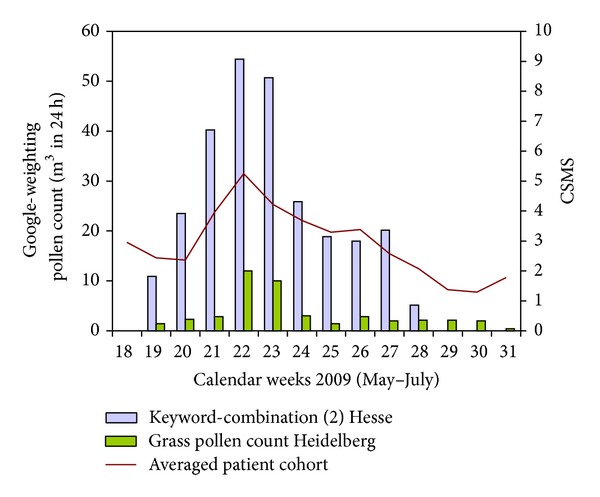
Course for Subgroup 1 (Wiesbaden study centre).

The value in the averaged Wiesbaden patient cohort for the keyword-combination (1 and 2) was a CCF of 0.766 (lag = −1, *P* < 0.001) (Table 2, Figure 5). The correlation strength of the grass pollen count data from the nearest pollen count station in Heidelberg was 0.495 (lag = 0, *P* < 0.001). In this population, a correlation strength of 0.752 (lag = −1, SE = 0.106, *P* < 0.01) was ascertained for the keyword-combination (2) in the “CSMS high” subgroup and a strength of 0.753 (lag = −1, SE = 0.106, *P* < 0.01) for the keyword-combination (1) in the “IgE high” subgroup. The comparative values for the pollen count data from Heidelberg for these two subgroups had a CCF of about 0.5.

### 3.4. Other Subgroup Analyses

**Figure 6:**
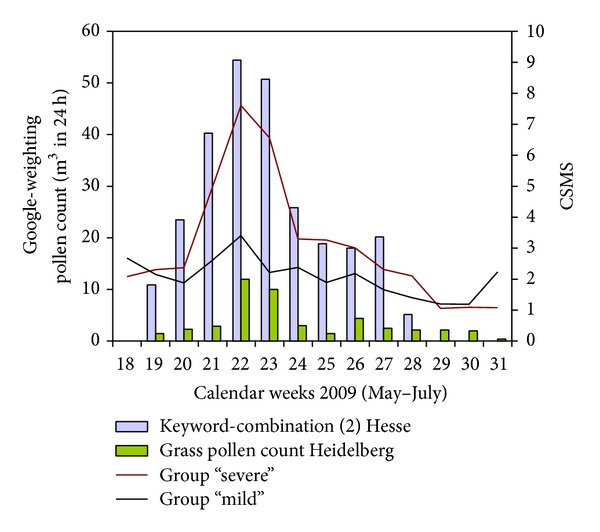
Course of the “severe” and “mild” group (Wiesbaden study centre).

Because of the significantly weaker correlation strengths in the subgroups with less allergic patients, additional “severe” (IgE ≥ class III (3.5 kU/L), CSMS ≥ 50% of the mean, asthma) and “mild” (IgE < class III (3.5 kU/L), CSMS < 50% of the mean, no asthma) groups were created. In all comparisons between these two groups at Aachen, Hamburg, and Wiesbaden centres, consistently higher CCF values were identified in the group of severely affected allergy sufferers for the keyword-combination (2). In the Wiesbaden centre, the highest superiority of the keyword-combination (2) could be observed.  Figure 6 shows the data evaluated for the averaged “severe” and “mild” group from the Wiesbaden centre. The keyword-combination (2) had a CCF of 0.808 (lag = −1) for the group “severe” (group “mild,” 0.616 CCF (lag = −1)). The corresponding grass pollen count data from Heidelberg showed a CCF of 0.553 (lag = −2).

## 4. Discussion

The results of the three centre analyses show moderate to strong correlations with the Google keyword-combination (up to >0.8 CCF) in 10 out of 11 groups (three averaged patient cohorts and the eight subgroups of severe allergy sufferers). The “asthma” (*n* = 2) group from Hamburg is the only exception with a CCF under 0.5. Thus, the correlation strengths of the Google data in these groups were invariably better than those of the grass pollen count data from the nearest pollen count stations. Other pooled group analyses with the severe allergy sufferers further strengthened the good results of the Google data.

Adjustment of the Google data (Google data (2)) due to the birch pollen season at the beginning of May showed an even stronger correlation. It is therefore possible to process the data later for certain pollen allergy sufferers.

The Hamburg study site, besides having the aforementioned outlier group of severe allergy sufferers, was the centre with the weakest correlations with the Google data. This centre showed extraordinary, erratic CSMS courses of the patients, which could be due on the one hand to a smaller patient data pool (*n* = 14) and on the other hand—compared to the other centres—to the highest TRMS.

### 4.1. Estimation of the Forecast

A negative lag of −1 was often observed for the Google data values. This could indicate that possible preventive measures were taken before pollen exposure, since the media often report the start of the pollen season or high pollen counts and allergy sufferers are possibly getting more information on the Internet. In addition, patient symptoms begin with the start of the pollen season and Internet queries could be made before the symptoms are strongest or before they become even stronger. This influence, known in the statistics as confounding, offers the possibility to determine the pollen count a day in advance. Therefore it offers the possibility to avoid or reduce the allergen exposure for all those who are suffering from pollen allergies and is thus supporting the most important factor in the therapeutic ranking.

### 4.2. Confounder

Since the patient's exact location was unknown, it was assumed that the patients were from the region surrounding the respective study centre. Based on the long-term design of the study (>1 year, 14 visits), however, this is to be expected. Because the Google data were generated according to state, one can also presume the direct correlation for this analysis.

### 4.3. Modern Data Analysis

The Google search engine has continued to improve during the past four years in that even more exact search results can be displayed based on the availability of more data. For example, in 2011, an analysis using the keyword-combination could be performed for all 16 states in Germany. In comparison, there were only nine analysable states in 2009. Based thereon and on the growth of worldwide Internet use [21], it is to be expected that an even more exact analysis of the pollen count can be conducted not for Germany alone. Regionally pooled Google data could therefore be used to derive correlations especially for pollen types that do not travel great distances.

### 4.4. Clinical Studies

The impact of pollen count on studies with pollinosis patients is well known and correlations have already been proven [17, 18]. The costs and expense for recruiting a suitable patient population are very high [22]. For the area of allergology, the results presented here can yield more validity of the study data. In the case of placebo-controlled studies, the data of placebo populations in particular can be examined and interpreted more precisely, especially in the context of the cohort receiving the study drug. Already completed studies as far back as 2004 could also be analysed in retrospect for differences in the symptom and medication scores of the study subjects based on freely available Google data.

The complexity of the pollen flight and the different clinical pictures of a person with pollinosis are of course difficult to be analysed with a general search engine based algorithm, as it is possible for other diseases. In our analysis we performed an algorithm for patients with a grass pollen allergy only.

## 5. Conclusion

Particularly for countries that do not have a dense network of pollen count stations, this Google-based algorithm would represent an alternative for determining pollen counts and even provide forecasts for the next day. It thus considers and supports the most important factor in the therapeutic approach to the clinical picture of pollinosis, namely, the avoidance of the allergen exposure. A prerequisite, of course, is the availability of an Internet infrastructure in the respective regions.

The correlations determined here are very clearly connected, but they must be further confirmed using a larger, transregional patient pool.

## Figures and Tables

**Table 1:** Results of cross-correlations for the Aachen study centre.

	Subgroup 1 Averaged CSMS (*n* = 16)				Subgroup 2 IgE ≥ class III (3.5 kU/L) (*n* = 8)				Subgroup 2 IgE < class III (3.5 kU/L) (*n* = 8)				Subgroup 4 CSMS ≥ 50% of mean (*n* = 13)				Subgroup 4 CSMS < 50% of mean (*n* = 3)			
	CCF	lag	SE	*P* value	CCF	lag	SE	*P* value	CCF	lag	SE	*P* value	CCF	lag	SE	*P* value	CCF	lag	SE	*P* value
**Grass pollen count Bonn**	0.466	−1	0.107	<0.001	0.441	6	0.110	<0.001	0.386	−1	0.107	<0.001	0.461	−1	0.107	<0.001	0.395	7	0.111	<0.001
**Grass pollen count Mönchengladbach**	0.630	0	0.107	<0.001	0.607	0	0.107	<0.001	0.412	−7	0.111	<0.001	0.622	0	0.107	<0.001	0.339	3	0.108	<0.01

**Keyword-combination (1)** NRW	0.643	−1	0.106	<0.001	0.517	−1	0.106	<0.001	**0.645**	**−1**	**0.106**	**<0.001**	0.607	−1	0.106	<0.001	**0.486**	**1**	**0.106**	**<0.001**
**Keyword-combination (2)** NRW	**0.715**	**−1**	**0.106**	**<0.001**	**0.608**	**−1**	**0.106**	**<0.001**	0.622	−1	0.106	<0.001	**0.688**	**−1**	**0.106**	**<0.001**	0.420	1	0.106	<0.001

**Table 2:** Results of cross-correlations for the Wiesbaden study centre.

	Subgroup 1 Averaged CSMS (*n* = 22)				Subgroup 2 IgE ≥ class III (3.5 kU/L) (*n* = 17)				Subgroup 2 IgE < class III (3.5 kU/L) (*n* = 5)				Subgroup 3 Asthma (*n* = 2)				Subgroup 3 No asthma (*n* = 20)				Subgroup 4 CSMS ≥ 50% of mean (*n* = 17)				Subgroup 4 CSMS < 50% of mean (*n* = 5)			
	CCF	lag	SE	*P* value	CCF	lag	SE	*P* value	CCF	lag	SE	*P* value	CCF	lag	SE	*P* value	CCF	lag	SE	*P* value	CCF	lag	SE	*P* value	CCF	lag	SE	*P* value
**Grass pollen count Heidelberg**	0.495	0	0.107	<0.001	0.492	−1	0.108	<0.001	0.321	−7	0.112	<0.01	0.574	−2	0.108	<0.001	0.441	0	0.107	<0.001	0.511	0	0.107	<0.001	0.300	−5	0.110	<0.01

**Keyword-combination (1)** HE	**0.766**	**−1**	**0.106**	**<0.001**	**0.753**	**−1**	**0.106**	**<0.001**	**0.352**	**−4**	**0.108**	**<0.01**	0.726	−1	0.105	<0.001	**0.685**	**−1**	**0.106**	**<0.001**	0.728	−1	0.106	<0.001	**0.524**	**−1**	**0.106**	**<0.001**
**Keyword-combination (2)** HE	**0.766**	**−1**	**0.106**	**<0.001**	0.748	−1	0.106	<0.001	0.336	−4	0.108	<0.01	**0.743**	**−1**	**0.106**	<0.001	0.680	−1	0.106	<0.001	**0.752**	**−1**	**0.106**	**<0.001**	0.400	−1	0.106	<0.001

## Abbreviations

AR: Allergic rhinitis
CCF: Cross-correlation coefficient
CSMS: Combined symptom medication score
HE: Hesse
NRW: North Rhine-Westphalia
RDBPC: Randomized, double-blind, placebo-controlled
RRTSS: Retrospective rhinoconjunctivitis total symptom score
RTSS: Rhinoconjunctivitis total symptom score
TRMS: Total rescue medication score.
